# Fluorescein Interferes With Hemoglobin Measurement During Intra-operative Point-of-Care Blood Gas Analysis: A Clinical Observation

**DOI:** 10.7759/cureus.108925

**Published:** 2026-05-15

**Authors:** Yuki Kojima, Takuya Moriwaki, Haruka Kitamura, Kazuhiko Nakano, Kazuya Hirabayashi

**Affiliations:** 1 Anesthesiology, Asahi General Hospital, Asahi, JPN; 2 Neurological Surgery, Asahi General Hospital, Asahi, JPN; 3 Clinical Services and Technology, Asahi General Hospital, Asahi, JPN; 4 Clinical Laboratory, Asahi General Hospital, Asahi, JPN

**Keywords:** blood gas analysis, fluorescein dye, hemoglobin, intra-operative, point-of-care test

## Abstract

We highlight a clinically important but under-recognized interference in hemoglobin measurement caused by intravenous fluorescein during neurosurgical procedures. A 70-year-old woman undergoing brain tumor resection showed normal initial blood gas results, but repeated point-of-care co-oximetry failed to measure hemoglobin after fluorescein administration. An alternative method enabled successful measurement of hemoglobin and transfusion guidance. Similar measurement failures were recorded previously, with failure in 7 of 12 cases. The fact that analyzers using different absorbance wavelengths were less affected suggests that spectral overlap between fluorescein and co-oximetry wavelengths may underlie the interference. A volunteer study further demonstrated that measurement failure occurred shortly after fluorescein administration but resolved within approximately 1 h. Clinicians should be aware of this limitation and consider alternative or confirmatory methods, such as central laboratory testing, when accurate hemoglobin assessment is critical.

## Editorial

We report a clinically important but under-recognized interference in point-of-care hemoglobin measurement following intravenous fluorescein (FLUORESCEITE, Novartis, Switzerland) administration during brain tumor surgery [1]. A 70-year-old woman underwent a craniotomy under general anesthesia for tumor resection. Initial arterial blood gas (ABG) analysis using RAPIDPoint 500e (SIEMENS, Germany) showed normal values for all measurable parameters. Following craniotomy, fluorescein was administered at the neurosurgeon’s request to aid intraoperative visualization. An 800-mL blood loss during tumor manipulation prompted a second ABG analysis. Although most parameters (e.g., electrolytes and oxygenation) were measurable, none of the hemoglobin-related parameters could be assessed. This issue persisted despite three repeated measurements. The Department of Clinical Laboratory suggested the use of an automated hematology analyzer (XN-1000; Sysmex, Japan). The analyzer revealed a hemoglobin level of 7.8 g/dL, prompting red blood cell transfusion. Surgery proceeded without further complications. In response to our inquiry, SIEMENS suggested that the improved sensitivity of newer co-oximetry systems in RAPIDPoint 500e may interpret altered absorbance patterns as unmeasurable values.

To investigate the interference in hemoglobin measurement, we retrospectively reviewed the anesthesia records of patients who underwent craniotomy with intraoperative fluorescein between August 2023 and April 2025 at the Asahi General Hospital. This phenomenon was observed in 7 of 12 neurosurgical cases involving fluorescein administration. In four cases, hemoglobin measurement using RAPIDPoint 500e failed post-administration. Notably, this issue was also observed in the previously used RAPIDLab 1200 series analyzer in three cases (Table 1).

**Table 1:** Characteristics and results of the cases included in this study Failure of hemoglobin-related parameters was observed in 7 of the 12 cases to undergo fluorescein administration at our institution.

Case number	Age	Sex	Height (cm)	Weight (kg)	Blood gas analyzer	Dose of fluorescein (mg)	Failure
1	62	Female	138	39	RAPIDLab 1200	790	-
2	64	Female	147	68	RAPIDLab 1200	500	+
3	76	Female	150	48	RAPIDLab 1200	1000	-
4	75	Female	165	44	RAPIDLab 1200	500	+
5	75	Male	170	71	RAPIDLab 1200	1500	+
6	82	Female	145	45	RAPIDPoint 500e	900	-
7	62	Female	166	67	RAPIDPoint 500e	1300	-
8	76	Female	149	33	RAPIDPoint 500e	700	+
9	69	Female	162	62	RAPIDPoint 500e	1000	-
10	69	Female	149	38	RAPIDPoint 500e	800	+
11	76	Female	149	34	RAPIDPoint 500e	700	+
12	84	Female	153	53	RAPIDPoint 500e	1000	+

Co-oximetry, used in both the RAPIDPoint and RAPIDLab series, employs multiple wavelengths to measure various hemoglobin species, including oxyhemoglobin, deoxyhemoglobin, carboxyhemoglobin, and methemoglobin. Although RAPIDPoint 500e offers higher accuracy, it may also be more susceptible to interference caused by dye solutions such as fluorescein. Unfortunately, when contacted, SIEMENS did not disclose the absorbance wavelengths of these devices, limiting their further interpretation. We also contacted Sysmex regarding why the XN-1000 used in the Department of Clinical Laboratory was able to obtain accurate hemoglobin measurements. They noted that the device does not use a co-oximetry system but relies on the SLS-hemoglobin detection method. XN-1000 uses an absorbance wavelength of 555 nm, distinct from the absorption peak of fluorescein at 494 nm; thus, successful measurements in this context may be attributed to this spectral difference. This observation aligns with previous findings in which dyes such as Patent Blue V interfered with co-oximetry and pulse oximetry by altering the absorbance spectra, thereby invalidating hemoglobin quantification [2-4]. In ophthalmology, fluorescein angiography and indocyanine green angiography affect complete blood count results [5-7]. de Groot reported that an alteration in fluorescence measurement in cells persists for at least 6 h after fluorescein angiography [5]. The increased fluorescence in lymphocytes, monocytes, and granulocytes may persist for up to 20 h following fluorescein injection [6].

We investigated the time course following fluorescein administration until hemoglobin measurement became feasible, using a healthy volunteer, a 38-year-old man (YK, one of the authors). An intravenous line was established with an 18-gauge catheter in the forearm of the volunteer (174 cm, 85 kg). The hemoglobin level was measured using both XN-1000 and RAPIDPoint 500e before fluorescein administration and every 30 min thereafter (Table 2). He received 1000 mg of fluorescein. While XN-1000 consistently enabled hemoglobin measurement, RAPIDPoint 500e failed to provide a measurement 30 min post-administration. By 60 min post-administration, the hemoglobin level was measurable with both devices. These findings suggest that fluorescein may cause similar interference with co-oximetry-based measurements. Even when results are generated, their reliability remains unverified owing to a lack of clinical validation. Moreover, inaccurate hemoglobin measurements can compromise intraoperative anesthetic management.

**Table 2:** Hemoglobin measurement after fluorescein administration A healthy 38-year-old man (174 cm, 85 kg) received 1000 mg of intravenous fluorescein. Blood samples were collected from a peripheral vein before administration and at 30, 60, 90, and 120 min post-administration. Hemoglobin level was measured with both analyzers. The XN-1000 analyzer consistently provided measurable hemoglobin levels. RAPIDPoint 500e failed to provide a measurement at 30 min but provided measurements at 60 min post-administration.

Time	Hemoglobin values (g/dL)	
	RAPIDPoint 500e	XN-1000 analyzer
Before start	14.6	14.1
30 min	Failure	13.6
60 min	13.6	13.4
90 min	14.8	13.7
120 min	14.6	13.4

Although fluorescein remains indispensable for tumor visualization and surgical precision, even in intracranial aneurysm surgery [8], the time required for dye metabolism and restoration of accurate co-oximetry values remains uncertain. Additionally, the failure was neither consistent in the cases nor dose-dependent according to our data. Therefore, anesthesiologists should be aware that fluorescein administration may temporarily impair the accuracy of co-oximetry-based parameters. Alternative or confirmatory methods, such as central laboratory hemoglobin measurement, should be considered during surgery when accurate hemoglobin monitoring is essential. Departments using intravenous fluorescein should also check on this phenomenon with the manufacturers of their ABG analysis machines. Future studies should investigate the time required for dye metabolism and accurate co-oximetry restoration. For example, our work could be enhanced by observing the effect of fluorescein being added in vitro to ABG samples; this might help compare the effects of a clinically relevant blood concentration of fluorescein on the hemoglobin concentration. Furthermore, as fluorescein undergoes glucuronidation in the liver following intravenous administration, the hemoglobin level should also be examined in patients with impaired liver function. This type of interference has not been widely reported. We highlight the importance of increasing awareness of this phenomenon and recommend the consideration of alternative methods when accurate hemoglobin monitoring is essential.
